# A multiple sclerosis‐like disorder in patients with *OPA1* mutations

**DOI:** 10.1002/acn3.323

**Published:** 2016-07-19

**Authors:** Patrick Yu‐Wai‐Man, Achillefs Spyropoulos, Holly J. Duncan, Joseph V. Guadagno, Patrick F. Chinnery

**Affiliations:** ^1^ Wellcome Trust Centre for Mitochondrial Research, Institute of Genetic MedicineNewcastle UniversityNewcastle upon TyneNE1 3BZUnited Kingdom; ^2^ Newcastle Eye CentreRoyal Victoria InfirmaryNewcastle upon TyneNE1 3BZUnited Kingdom; ^3^ NIHR Biomedical Research Centre at Moorfields Eye Hospital and UCL Institute of OphthalmologyLondonEC1V 2PDUnited Kingdom; ^4^ Department of NeurologyRoyal Victoria InfirmaryNewcastle upon TyneNE1 4LPUnited Kingdom; ^5^ MRC‐Mitochondrial Biology UnitCambridge Biomedical CampusCambridgeCB2 0XYUnited Kingdom; ^6^ Department of Clinical NeurosciencesCambridge Biomedical CampusUniversity of CambridgeCambridgeCB2 0QQUnited Kingdom

## Abstract

We describe three unrelated patients presenting with a spinal cord syndrome and neuroimaging features consistent with multiple sclerosis (MS). All harbored a pathogenic *OPA1* mutation. Although the neurological phenotype resembled neuromyelitis optica (NMO), anti‐aquaporin 4 antibodies were not detected and the disorder followed a slow progressive course. The coincidental occurrence of *OPA1* mutations and an MS‐like disorder is likely to have modulated the phenotypic manifestations of both disorders, but unlike the previously reported association of Leber hereditary optic neuropathy and MS (Harding disease), the optic neuropathy in patients with *OPA1* mutations and an MS‐like disorder can be mild with a good visual prognosis.

## Introduction

Mutations in *OPA1* were first described in pedigrees with autosomal‐dominant optic atrophy (DOA), which is the most common form of inherited optic nerve blindness in the population.^1^, ^2^ The visual loss in DOA develops in early childhood and it has a relatively indolent course with 10–20% of *OPA1* mutation carriers remaining visually asymptomatic.^3^ Nonpenetrance is also well described, which contributes to the lack of a clear‐cut family history in some affected probands.

*OPA1* encodes for an inner mitochondrial membrane protein that regulates a host of important functions, including the balance between mitochondrial fusion and fission, the stability of the mitochondrial respiratory chain complexes, and the controlled release of proapoptotic cytochrome *c* molecules sequestered within the mitochondrial cristae.^4^ DOA is therefore a mitochondrially determined optic neuropathy characterized by the preferential loss of retinal ganglion cells within the inner retina, similar to Leber hereditary optic neuropathy (LHON).

In 1992, Anita Harding and colleagues described eight female patients with the m.11778A>G mitochondrial DNA (mtDNA) mutation who developed features of both LHON and multiple sclerosis (MS).^5^ Subsequent reports of MS occurring in association with all three primary LHON mutations (m.3460A>G, m.11778A>G, and m.14484T>C), raised the distinct possibility of a mechanistic link between these two disorders,^6^, ^7^, ^8^, ^9^ supported by mounting evidence of mitochondrial dysfunction in both acute and chronic MS lesions.^10^, ^11^, ^12^ Unlike classical demyelinating optic neuritis, visual loss in LHON‐MS cases (also known as Harding disease) is frequently bilateral at onset and the prognosis is poor with over half of all patients being registered legally blind.^13^ However, unlike the well‐recognized link between LHON mtDNA mutations and demyelination, the link between *OPA1* mutations and MS is not well established. To date, an MS‐like illness has only been described in one patient with an *OPA1* mutation,^14^ although another patient had coincidental MRI findings consistent with MS,^15^ and several case series have described abnormal white matter high signal changes in *OPA1* mutation carriers.^16^, ^17^, ^18^ In this report, we describe three additional unrelated white Caucasian patients who presented to our tertiary neuro‐ophthalmological center with a clinically definite spinal cord MS phenotype, and who were eventually found to harbor pathogenic *OPA1* mutations.

## Subjects

### Patient A

**Figure 1:**
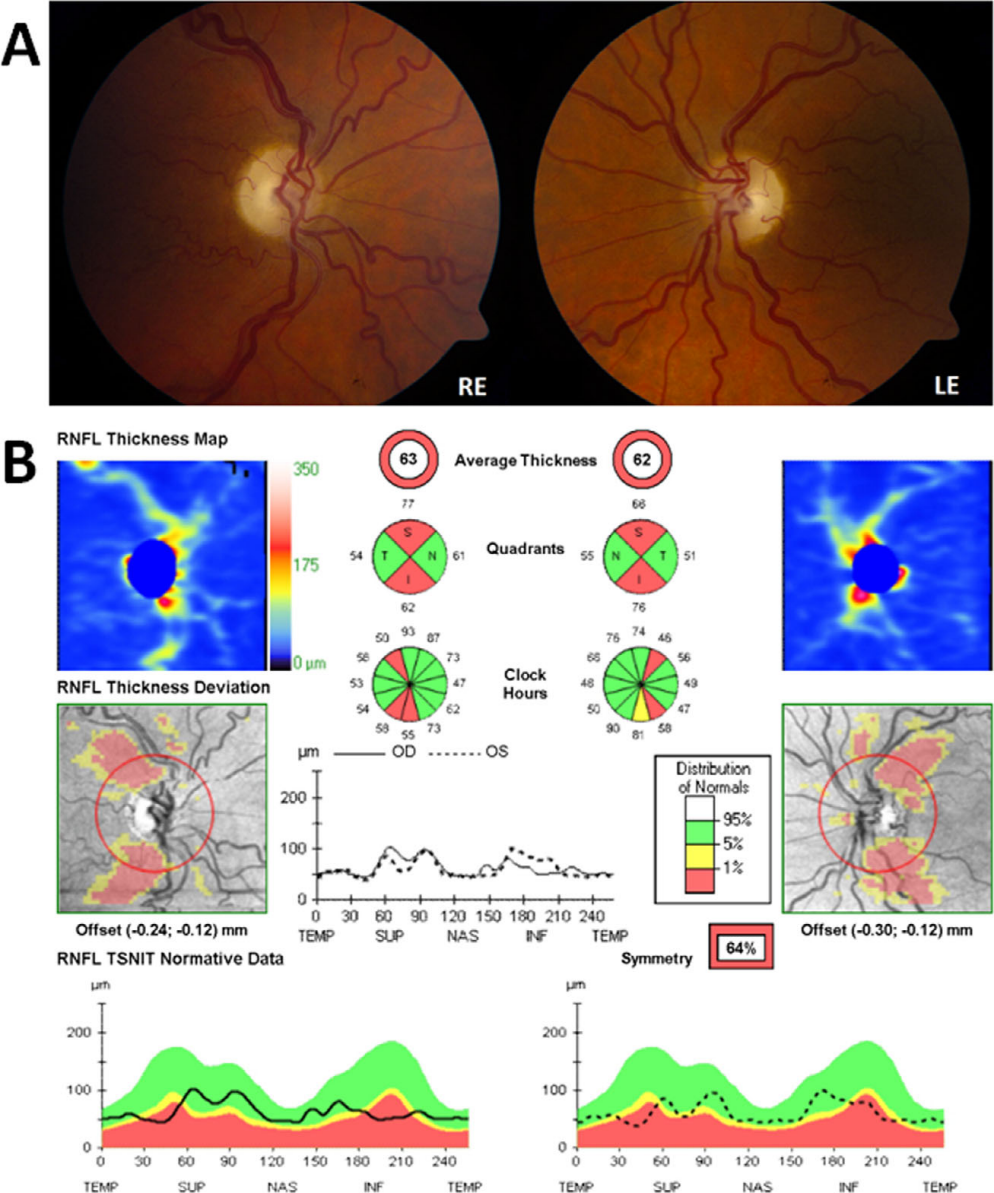
Optic discs appearance and optical coherence tomography (OCT) findings for Patient A. (A) Bilateral optic atrophy (RE, right eye; LE, left eye); (B) OCT measurements were obtained with the high‐resolution spectral‐domain Cirrus^TM^ platform (Carl Zeiss Meditec, Dublin, CA). The average peripapillary retinal nerve fiber layer thickness (RNFL) was 63 *μ*m in the right eye (OD) and 62 *μ*m in the left eye (OS). The analysis software automatically selects the appropriate normative range for the patient and the peripapillary RNFL measurements (dark traces) are represented within color‐coded distribution centiles (bottom panel): (1) red < 1%, (2) yellow 1–5%, and (3) green 5–95%.

**Figure 2:**
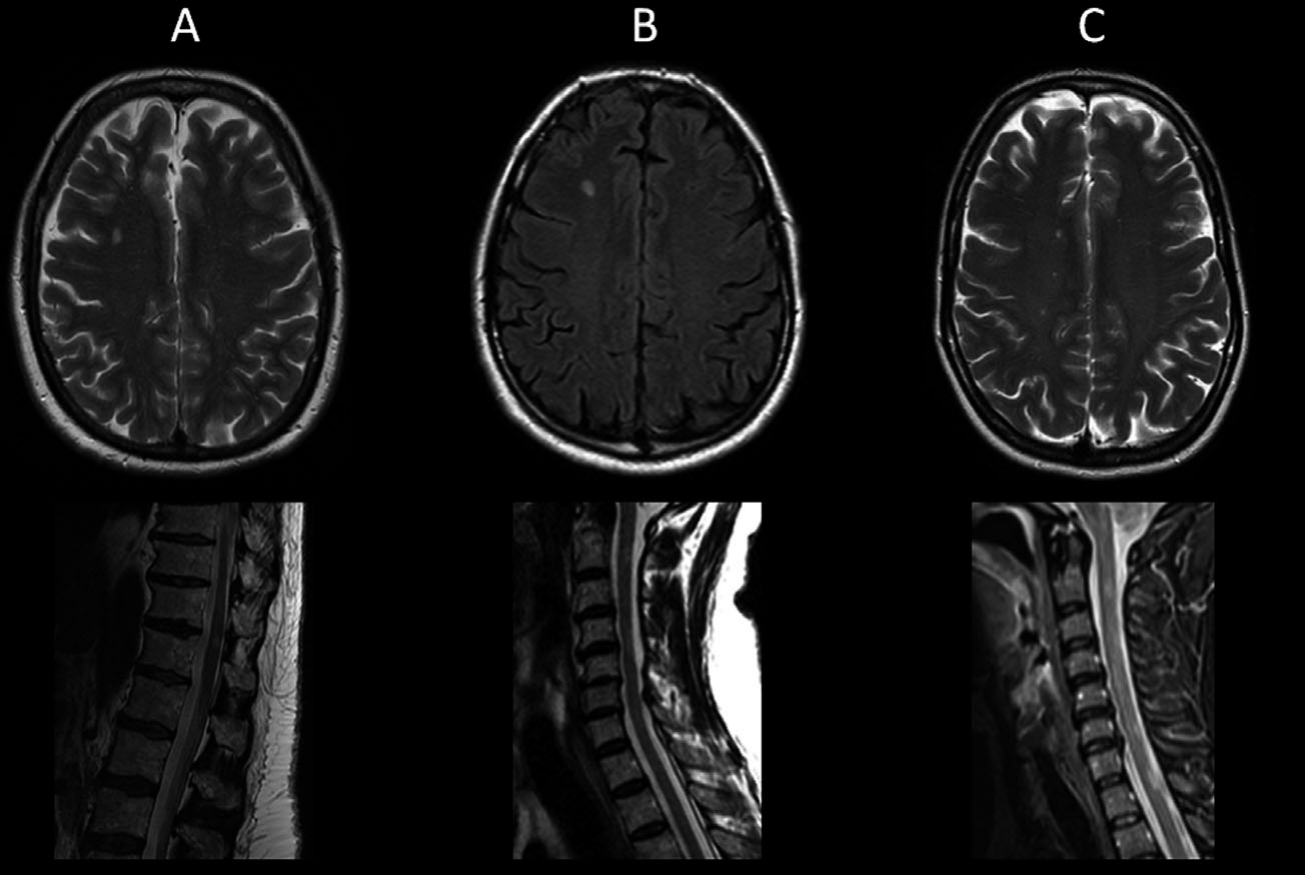
Brain and spinal cord MRI abnormalities in three patients with a multiple sclerosis‐like disorder and *OPA1* mutations. Multiple foci of T2 hyperintensity were observed in the periventricular and pericallosal white matter regions. Discreet areas of signal abnormalities were also present in the cervical spinal cord, none of which were longitudinally extensive. Taken together, these neuroradiological findings met the MRI diagnostic criteria for multiple sclerosis. Panel A is patient A, panel B is patient B, and panel C is patient C. Upper panel A = axial T2‐weighted imaging, lower panel A = sagittal T2‐weighted imaging. Upper panel B = axial T1‐weighted imaging, lower panel B = sagittal T2‐weighted imaging. Upper panel C = axial T2‐weighted imaging, lower panel C = sagittal T2‐weighted imaging.

A 60‐year‐old woman presented with a 4‐year history of worsening gait and sensory disturbance that was accompanied by urinary urgency and nocturia. She had noticed a slow deterioration in her vision from the age of 50 years, but she was still able to drive. Her mother was registered blind in her later years and she also suffered from Parkinson disease with dementia. The patient had two unaffected sisters. On examination, she had a spastic gait. Power in the lower limbs was normal, but there was increased tone and sustained ankle clonus. Reflexes were brisk and plantar responses were flexor. The patient had a mild reduction in visual acuity and dyschromatopsia, but there was no detectable visual field defect and pupillary light reflexes were preserved (Table 1). Optic disc pallor was noted on dilated fundus examination (Fig. 1). MRI of the brain and spinal cord revealed multiple foci of high T2 signal that were in keeping with demyelination (Fig. 2A). Oligoclonal bands were detected in the patient's cerebrospinal fluid (CSF). Routine hematological, biochemical, and immunological blood tests were normal, including folate and vitamin B12. Anti‐aquaporin 4 antibodies were not present in the patient's serum. Genetic testing for the three primary mtDNA LHON mutations was negative. The family history and atypical presentation led us to request *OPA1* genetic screening, which identified a novel heterozygous splice site mutation, c.2356‐1G>T. No clinical improvement was observed after an empirical 5‐day course of intravenous methylprednisolone. Although the patient's vision has remained stable, her spastic paraparesis has progressed rapidly over the subsequent 3 years of follow‐up (EDSS 6.0).

**Table 1:** Clinical and molecular characteristics of the three patients with *OPA1* mutations and an MS‐like disorder

Subject	Age	Sex	*OPA1* mutation	BCVA (Snellen)		Ishihara plates (/15)		Humphrey^TM^ fields (MD)		Average RNFL thickness (*μ*m)^a^	
				RE	LE	RE	LE	RE	LE	RE	LE
Patient A	60	F	c.2356‐1G>T	20/30	20/30	3	3	−0.74	−0.87	63	62
Patient B	56	M	c.870+5G>A	CF	CF	0	0	N/A^b^	N/A^b^	64	65
Patient C	39	F	c.870+5G>A	20/60	20/60	1	1	−1.22	−3.14	65	64

a Average peripapillary retinal nerve fiber layer thickness was measured using the high‐resolution spectral‐domain Cirrus^TM^ platform (Carl Zeiss Meditec, Dublin, CA).

b Patient B was unable to perform Humphrey^TM^ visual field perimetry. Goldmann fields showed bilateral dense central scotomas.

BCVA, best‐corrected visual acuity; CF, counting fingers; LE, left eye; MD, mean deviation (dB); RE, right eye; RNFL, retinal nerve fiber layer.

### Patient B

A 56‐year‐old man was referred with an 8‐year history of progressive walking difficulties with stiffness and weakness in his lower limbs, increasing balance difficulties and paraesthesiae in both hands. His visual difficulties became apparent at the age of 7 years and he has been registered legally blind (Table 1). Both optic discs were globally pale and optical coherence tomography (OCT) imaging showed significant thinning of the retinal nerve fiber layer (Fig. S1). The patient had marked asymmetric spastic paraparesis, with the left lower limb being more severely affected than the right. Reflexes were exaggerated at the knees and hypoactive at the ankles. He had reduced sensation to pinprick in a glove and stocking distribution, and vibration sense was absent distally in all four limbs. Neuroimaging was highly suggestive of inflammatory demyelination (Fig. S2B). The patient did not have a lumbar puncture. A comprehensive blood panel was normal and anti‐aquaporin 4 antibodies were not detected. *OPA1* genetic testing revealed a previously reported pathogenic heterozygous splice site mutation, c.870+5G>A. No clinical response was obtained after a three‐day course of high‐dose intravenous methylprednisolone. His EDSS changed quickly between 2012 and 2015 to 6.0. Interestingly, we subsequently heard that the patient's son had been diagnosed with relapsing‐remitting MS and he was receiving disease‐modifying therapy. The son did not harbor the *OPA1* mutation that was identified in his father.

### Patient C

A 39‐year‐old woman first presented in 2002 with paraesthesiae affecting both upper limbs, which lasted for 4 weeks before recovering fully. She represented in 2012 with bilateral numbness and paraesthesiae progressing from her feet to her knees over 1 week. There was accompanying tingling of the hands and positive L'Hermitte's phenomenon. She complained of gradually worsening vision from her early teenage years with no episodes of acute deterioration (Table 1). On dilated fundus examination, bilateral optic disc pallor was noted (Fig. S2). Her mother and a maternal uncle were also known to have bilateral optic atrophy, but with no overt neurological deficits. The rest of the patient's neurological examination was normal except for reduced vibration sense to the knee (EDSS 2.5). MRI of the brain and spinal cord revealed disseminated white matter lesions consistent with demyelination (Fig. 2C). CSF was not examined. The patient tested negative for anti‐aquaporin 4 antibodies and the three primary mtDNA LHON mutations (m.3460A>G, m.11778A>G, and m.14484T>C). Given the family history, *OPA1* genetic testing was requested and a heterozygous splice site mutation, c.870+5G>A, was detected.

## Discussion

The patients described in our case series fulfilled the revised McDonald criteria for a diagnosis of MS, with patients A and B having a primary progressive course, and patient C having a relapsing‐remitting course.^19^ All three patients harbored *OPA1* splice site mutations: the previously described c.870+5G>A variant, and a novel c.2356‐1G>T variant that affects the canonical splice acceptor site. The *OPA1* c.870+5G>A mutation causes early‐onset visual loss with a mean age of onset of 10 years old and visual acuities ranging from 20/30 to counting fingers.^3^ Two patients with this specific splice site mutation have also been reported where the optic atrophy was complicated by myopathy and peripheral neuropathy, but with no evidence of demyelination.^15^ MS affects an estimated 2 in 1000 people in the United Kingdom,^20^ and the prevalence of *OPA1*‐positive DOA is 2.9 per 100,000 in the North of England.^21^ Approximately, 1 in 18 million of the population or 3–4 people in the UK, will therefore be expected to have both an MS‐like disorder and carry *OPA1* mutations purely by chance. This would explain a case we previously described of a 58‐year‐old woman with an *OPA1* splice site mutation within intron 25 (c.2613+1G>A) who was unexpectedly found to have MRI findings consistent with demyelination, in the absence of any clear neurological deficits.^15^

Although the three patients we describe here are likely to be a coincidental occurrence, their clinical presentations are not entirely classical for either MS or DOA. As *OPA1* mutation carriers, they had an underlying predisposition to develop an optic neuropathy which progressed slowly over time, and all three patients developed a spinal cord syndrome.^22^ Unlike Patient B and Patient C who became aware of progressive visual deterioration in childhood, Patient A had an unusual late presentation in her early fifties with only a mild reduction in central vision (20/30 in both eyes). Although the sensory symptoms resolved in Patient C, suggestive of an acute inflammatory attack, the spinal cord syndromes were relentlessly progressive in the remaining two patients, with a relatively rapid deterioration compared to typical primary progressive multiple sclerosis. This would usually imply ongoing lingering inflammatory disease, but there was no response to high‐dose intravenous methylprednisolone in any of the patients described here. The most likely explanation is an accelerated neurodegenerative mechanism probably mediated by mitochondrial dysfunction. Bilateral visual failure with a spinal cord syndrome suggests NMO, but our patients were aquaporin‐4 antibody negative, making this diagnosis unlikely. Anti‐MOG antibody testing was, however, not performed.

The MS‐DOA overlap syndrome that we describe here seems distinct from the well‐established association between LHON and MS.^5^, ^6^, ^7^, ^8^, ^9^ With over 60 cases described to date, patients with LHON‐MS typically develop bilateral visual failure that is severe, irreversible, and punctuated by recurrent episodes of acute or subacute deterioration.^13^ Although a spinal cord presentation has been described, this is an infrequent occurrence. The majority of patients with LHON‐MS present with sequential visual loss and the predominant neurological phenotype follows a relapsing‐remitting course.^13^ These contrasting observations are intriguing because both LHON and DOA are mitochondrial optic neuropathies that primarily target the retinal ganglion cells, resulting in a monosymptomatic ocular disease.^23^ However, the spectrum of *OPA1* disease is not limited to the optic nerve and about 20% of mutation carriers can develop additional features, in particular, chronic progressive external ophthalmoplegia, ataxia, and peripheral neuropathy.^15^ In comparison, these classical multisystemic features of mitochondrial disease are rare in LHON.^23^ Different disease mechanisms are therefore likely to be involved in the development of the more severe neurological “plus” features in *OPA1* and LHON mtDNA mutation carriers. The characteristic vulnerability of the spinal cord seen in the three patients in our case series further highlights the complexity, and the limits of our knowledge, in fully understanding the pathological pathways that modulate mitochondrial neurodegeneration in MS. Clinicians should consider the possibility of an *OPA1*‐related MS‐like disorder in a patient presenting with progressive visual failure and neuroimaging features indicative of a more disseminated inflammatory process. This could lead to the identification of a pathogenic *OPA1* mutation, which will have important implications for the rest of the family and require appropriate genetic counseling. It would also allow the clinician to give a more guarded visual prognosis than would normally be the case in a typical case of demyelinating optic neuritis associated with MS.

## Conflicts of Interest

None declared.

## Supporting information


**Figure S1.** Optic discs appearance and OCT findings for Patient B. (A) Bilateral optic atrophy (RE, right eye; LE, left eye). (B) Optical coherence tomography (OCT) measurements were obtained with the high‐resolution spectral‐domain Cirrus^TM^ platform (Carl Zeiss Meditec, Dublin, CA). The average retinal nerve fiber layer thickness was 64 *μ*m in the right eye (OD) and 65 *μ*m in the left eye (OS).
**Figure S2.** Optic discs appearance and OCT findings for Patient C. (A) Bilateral optic atrophy (RE, right eye; LE, left eye). (B) Optical coherence tomography (OCT) measurements were obtained with the high‐resolution spectral‐domain Cirrus^TM^ platform (Carl Zeiss Meditec, Dublin, CA). The average retinal nerve fiber layer thickness was 65 *μ*m in the right eye (OD) and 64μm in the left eye (OS).

## References

[1] Delettre C , Lenaers G , Pelloquin L , et al. OPA1 (Kjer type) dominant optic atrophy: a novel mitochondrial disease. Mol Genet Metab 2002;75:97–107. 11855928 10.1006/mgme.2001.3278

[2] Alexander C , Votruba M , Pesch UEA , et al. OPA1, encoding a dynamin‐related GTPase, is mutated in autosomal dominant optic atrophy linked to chromosome 3q28. Nat Genet 2000;26:211–215. 11017080 10.1038/79944

[3] Yu‐Wai‐Man P , Griffiths PG , Burke A , et al. The prevalence and natural history of dominant optic atrophy due to opa1 mutations. Ophthalmology 2010;117:1538–1546. 20417570 10.1016/j.ophtha.2009.12.038 PMC4040407

[4] Burte F , Carelli V , Chinnery PF , Yu‐Wai‐Man P . Disturbed mitochondrial dynamics and neurodegenerative disorders. Nat Rev Neurol 2015;11:11–24. 25486875 10.1038/nrneurol.2014.228

[5] Harding AE , Sweeney MG , Miller DH , et al. Occurrence of a multiple sclerosis‐like illness in women who have a lebers hereditary optic neuropathy mitochondrial‐dna mutation. Brain 1992;115:979–989. 1393514 10.1093/brain/115.4.979

[6] Bhatti MT , Newman NJ . A multiple sclerosis‐like illness in a man harboring the mtDNA 14484 mutation. J Neuroophthalmol 1999;19:28–33. 10098545

[7] Horvath R , Abicht A , Shoubridge EA , et al. Leber's hereditary optic neuropathy presenting as multiple sclerosis‐like disease of the CNS. J Neurol 2000;247:65–67. 10701903 10.1007/s004150050015

[8] Kovacs GG , Hoftberger R , Horvath R , et al. Neuropathology of white matter disease in Leber's hereditary optic neuropathy. Brain 2005;128:35–41. 15483043 10.1093/brain/awh310

[9] La Russa A , Cittadella R , Andreoli V , et al. Leber's hereditary optic neuropathy associated with a multiple‐sclerosis‐like picture in a man. Multiple Sclerosis J 2011;17:763–766. 10.1177/1352458511404033 21685233

[10] Andrews H , White K , Thomson C , Edgar J , Bates D , Griffiths I , Turnbull D , Nichols P . Increased axonal mitochondrial activity as an adaptation to myelin deficiency in the Shiverer mouse. J Neurosci Res 2006; 83: 1533–9. 16555298 10.1002/jnr.20842

[11] Mahad D , Ziabreva I , Lassmann H , Turnbull D . Mitochondrial defects in acute multiple sclerosis lesions. Brain 2008;131:1722–1735. 18515320 10.1093/brain/awn105 PMC2442422

[12] Mahad DJ , Ziabreva I , Campbell G , et al. Mitochondrial changes within axons in multiple sclerosis. Brain 2009;132:1161–1174. 19293237 10.1093/brain/awp046 PMC3605917

[13] Pfeffer G , Burke A , Yu‐Wai‐Man P , et al. Clinical features of MS associated with Leber hereditary optic neuropathy mtDNA mutations. Neurology 2013;81:2073–2081. 24198293 10.1212/01.wnl.0000437308.22603.43 PMC3863351

[14] Verny C , Loiseau D , Scherer C , et al. Multiple sclerosis‐like disorder in OPA1‐related autosomal dominant optic atrophy. Neurology 2008;70(13 Pt 2):1152–1153. 18287570 10.1212/01.wnl.0000289194.89359.a1

[15] Yu‐Wai‐Man P , Griffiths PG , Gorman GS , et al. Multi‐system neurological disease is common in patients with OPA1 mutations. Brain 2010;133(Pt 3):771–786. 20157015 10.1093/brain/awq007 PMC2842512

[16] Carelli V , Musumeci O , Caporali L , et al. Syndromic parkinsonism and dementia associated with OPA1 missense mutations. Ann Neurol 2015;78:21–38. 25820230 10.1002/ana.24410 PMC5008165

[17] Manners DN , Rizzo G , La Morgia C , et al. Diffusion tensor imaging mapping of brain white matter pathology in mitochondrial optic neuropathies. AJNR Am J Neuroradiol 2015;36:1259–1265. 25792533 10.3174/ajnr.A4272 PMC7965282

[18] Rocca MA , Bianchi‐Marzoli S , Messina R , et al. Distributed abnormalities of brain white matter architecture in patients with dominant optic atrophy and OPA1 mutations. J Neurol 2015;262:1216–1227. 25794858 10.1007/s00415-015-7696-5

[19] Polman CH , Reingold SC , Banwell B , et al. Diagnostic criteria for multiple sclerosis: 2010 revisions to the mcdonald criteria. Ann Neurol 2011;69:292–302. 21387374 10.1002/ana.22366 PMC3084507

[20] Mackenzie IS , Morant SV , Bloomfield GA , et al. Incidence and prevalence of multiple sclerosis in the UK 1990‐2010: a descriptive study in the general practice research database. J Neurol Neurosurg Psychiatry 2014;85:76–84. 24052635 10.1136/jnnp-2013-305450 PMC3888639

[21] Yu‐Wai‐Man P , Chinnery PF . Dominant optic atrophy: novel OPA1 mutations and revised prevalence estimates. Ophthalmology 2013; 120: 1712–1721. 23916084 10.1016/j.ophtha.2013.04.022 PMC6542663

[22] Compston A , Coles A . Multiple sclerosis. Lancet 2008;372:1502–1517. 18970977 10.1016/S0140-6736(08)61620-7

[23] Carelli V , Ross‐Cisneros FN , Sadun AA . Mitochondrial dysfunction as a cause of optic neuropathies. Prog Retin Eye Res 2004;23:53–89. 14766317 10.1016/j.preteyeres.2003.10.003

