# Supplementary material for: A multiple sclerosis‐like disorder in patients with OPA1 mutations
Source: Ann Clin Transl Neurol. 2016 Jul 19;3(9):723–9. doi: 10.1002/acn3.323 (PMC5018584; doi:10.1002/acn3.323)
Supplement: Supplementary file 1 — Figure S1. Optic discs appearance and OCT findings for Patient B. (A) Bilateral optic atrophy (RE, right eye; LE, left eye). (B) Optical coherence tomography (OCT) measurements were obtained with the high‐resolution spectral‐domain CirrusTM platform (Carl Zeiss Meditec, Dublin, CA). The average retinal nerve fiber layer thickness was 64 μm in the right eye (OD) and 65 μm in the left eye (OS). Figure S2. Optic discs appearance and OCT findings for Patient C. (A) Bilateral optic atrophy (RE, right eye; LE, left eye). (B) Optical coherence tomography (OCT) measurements were obtained with the high‐resolution spectral‐domain CirrusTM platform (Carl Zeiss Meditec, Dublin, CA). The average retinal nerve fiber layer thickness was 65 μm in the right eye (OD) and 64μm in the left eye (OS). [file ACN3-3-723-s001.pdf]

# Supplementary Figure 1

A

B

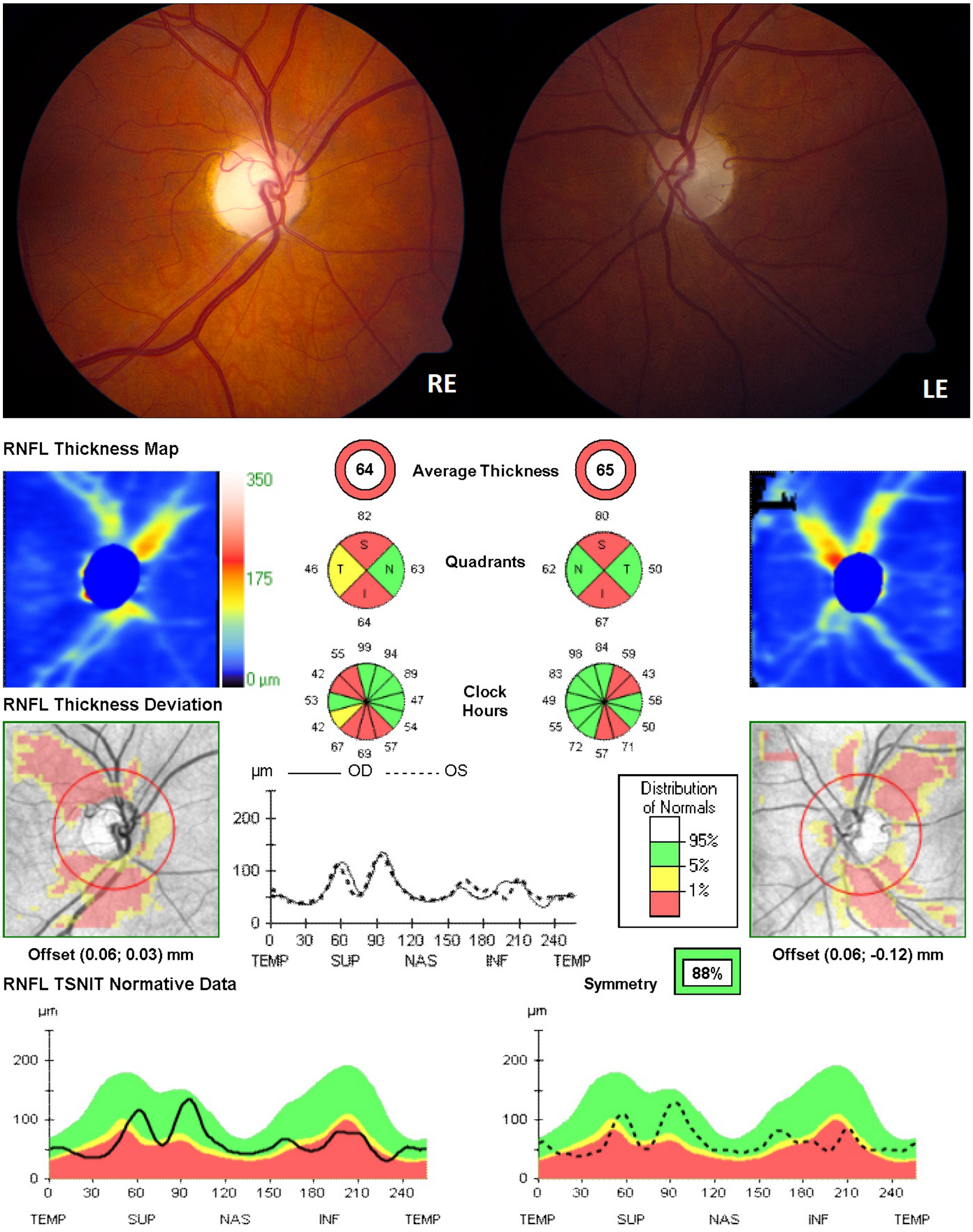


**Optic discs appearance and OCT findings for Patient B**

(A)Bilateral optic atrophy (RE = right eye, LE = left eye). (B) Optical coherence tomography (OCT) measurements were obtained with the high-resolution spectral-domain Cirrus$^{TM}$ platform (Carl Zeiss Meditec, Dublin, CA). The average retinal nerve fibre layer thickness was 64µm in the right eye (OD) and 65µm in the left eye (OS)

# Supplementary Figure 2

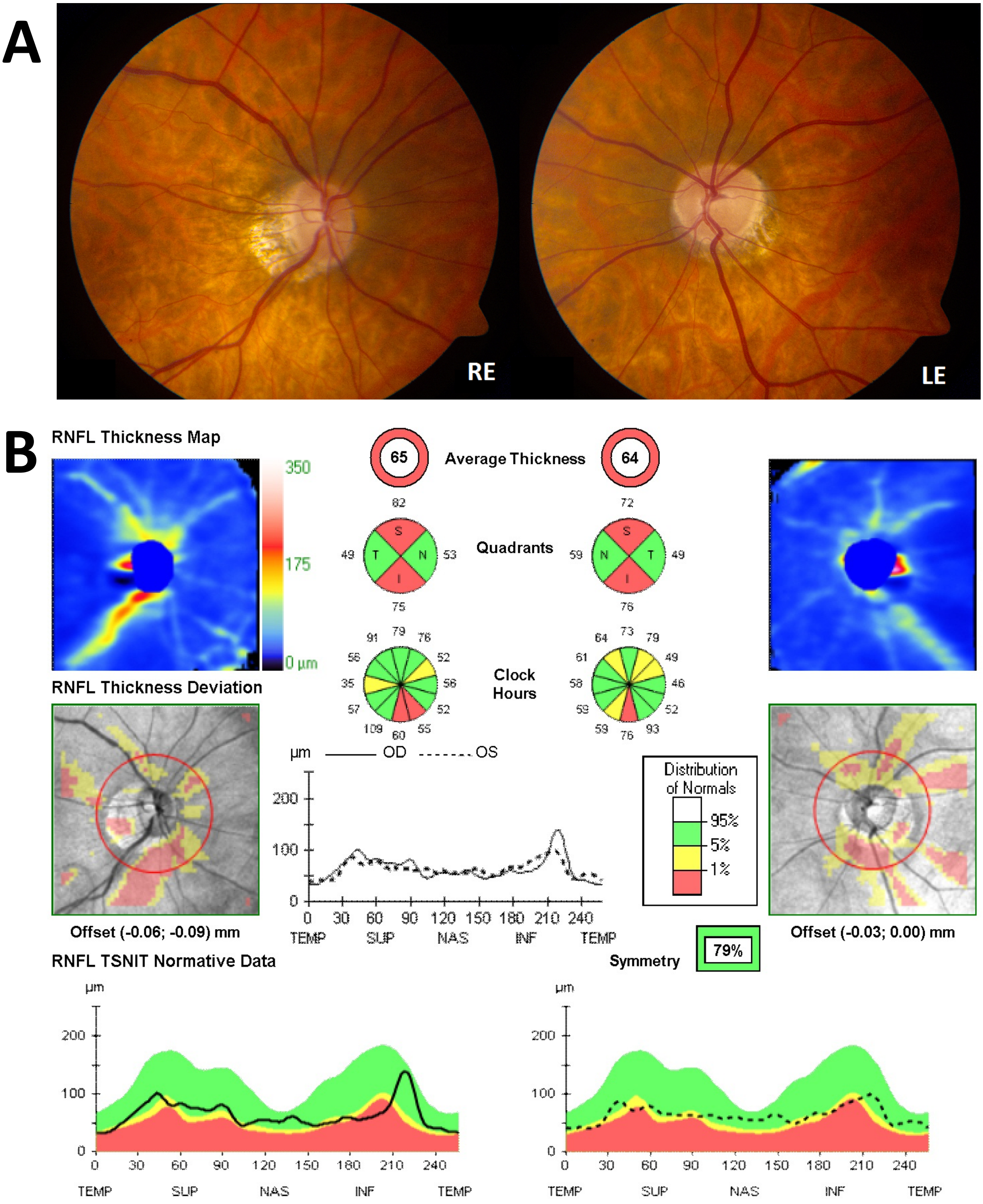


**Optic discs appearance and OCT findings for Patient C**

(A)Bilateral optic atrophy (RE = right eye, LE = left eye). (B) Optical coherence tomography (OCT) measurements were obtained with the high-resolution spectral-domain Cirrus$^{TM}$ platform (Carl Zeiss Meditec, Dublin, CA). The average retinal nerve fibre layer thickness was 65µm in the right eye (OD) and 64µm in the left eye (OS)
